# Utility of posaconazole therapeutic drug monitoring and assessment of plasma concentration threshold for effective prophylaxis of invasive fungal infections: a meta-analysis with trial sequential analysis

**DOI:** 10.1186/s12879-018-3055-3

**Published:** 2018-04-02

**Authors:** Lu Chen, Yan Wang, Tao Zhang, Ying Li, Ti Meng, Leichao Liu, Ruifang Hao, Yalin Dong

**Affiliations:** Department of Pharmacy, The First Affiliated Hospital of Xi’an Jiaotong University, Xi’an, 710061 China

**Keywords:** Posaconazole, Prophylaxis, Target plasma concentration, Invasive fungal infections, Therapeutic drug monitoring

## Abstract

**Background:**

Posaconazole therapeutic drug monitoring (TDM) is increasingly used in clinical practice. However, the utility of posaconazole TDM and the target of posaconazole plasma concentration for clinical successful prophylaxis remain uncertain and controversial. The aim of this study was to evaluate posaconazole exposure-response relationship and determine an optimum posaconazole concentration for prophylaxis against invasive fungal infections (IFIs).

**Methods:**

Bibliographic databases were searched (from inception to September 2017) to select studies including the clinical outcomes below and above concentration cut-off value of 0.5 mg/L and 0.7 mg/L. The reliability of the results were evaluated with trial sequential analysis (TSA).

**Results:**

Twenty-eight studies with 1930 patients included were analyzed. The results of our pooled analysis demonstrated that patients with posaconazole plasma concentrations over 0.5 mg/L were twice more likely to achieve successful responses compared with those with lower concentrations (odds ratio, OR = 1.98, 95% confidence interval, CI 1.09–3.58, *P* = 0.02) while the threshold, 0.7 mg/L showed no significant difference (OR = 1.84, 95% CI 0.94–3.63, *P* = 0.08). The TSA results showed that there was sufficient information to support these findings.

**Conclusions:**

An optimal posaconazole concentration target of 0.5 mg/L is suggested to ensure the clinical prophylactic efficacy and may help reduce the dosage and dose-dependent toxicity comparing with the target of 0.7 mg/L.

**Electronic supplementary material:**

The online version of this article (10.1186/s12879-018-3055-3) contains supplementary material, which is available to authorized users.

## Background

Invasive fungal infections (IFIs) are substantial causes of morbidity and mortality in immunocompromised hosts, such as patients with hematological malignancies and solid-organ transplant recipients [1]. Prophylaxis was widely accepted as an important intervention in this setting [2]. Posaconazole is a second-generation triazole agent with antifungal activity against a wide range of yeasts (candida species) and molds (*Aspergillus* species, *Zygomycetes*, and *Fusarium* species) [3, 4]. It has been strongly recommended as a prophylaxis of IFIs by guidelines from IDSA and ESCMID with high-quality evidence [5–7]. The US Food and Drug Administration (FDA) have approved three formulations, including the oral suspension, the recently delayed-release tablet and intravenous formulations. Due to the large interindividual variability in bioavailability and drug-drug interactions, therapeutic drug monitoring (TDM) is advised by IDSA and FDA in order to ensure adequate exposure and optimize clinical efficacy for posaconazole suspension [5, 8, 9].

The growing studies reported that there is a significant exposure-response relationship between posaconazole plasma concentrations and prophylactic efficacy [10–12]. Posaconazole TDM is also increasingly used in clinical practice to achieve a plasma concentration target of 0.5 mg/L at steady state which is equivalent to the minimum inhibitory concentration (MIC_90_) of posaconazole for most *Aspergillus spp*. and was also recommended by the 4th European Conference on Infections in Leukaemia (ECIL-4) [13]. Thus, a stable drug concentration at 0.5 mg/L has been suggested in several posaconazole TDM studies [14–17]. Nevertheless, Jang et al. recommended a target at 0.7 mg/L which was also adopted in FDA document [8, 12]. Meanwhile, posaconazole showed a good long-term safety profile compared with voriconazole and itraconazole [18–20]. Therefore, the utility of posaconazole TDM remains a controversial issue and most of related studies are limited by single-center practice and small sample size.

Although exposure-response relationship has been examined in several studies, it is still unclear whether TDM should be routinely performed during the process of posaconazole prophylaxis. Furthermore, there was no final consensus reached about posaconazole concentration target for prophylactic use to date. The aim of this study was to assess the relationship between posaconazole plasma concentration and clinical prophylactic efficacy and to define the optimum posaconazole concentration based on a meta-analysis.

## Methods

### Search strategy

We conducted a literature search in PubMed, EMBASE from inception to September 2017. A complementary manual literature search was performed by checking the reference lists in identified studies editorials, and related reviews. The ‘key words’ used were posaconazole, noxafil, concentration, exposure, efficacy, response, drug monitoring, pharmacovigilance, drug-related side effects and adverse reactions, drug eruptions. All searches were limited to human studies (see the detailed searching strategy in the Additional file 1).

### Selection criteria

**Fig. 1:**
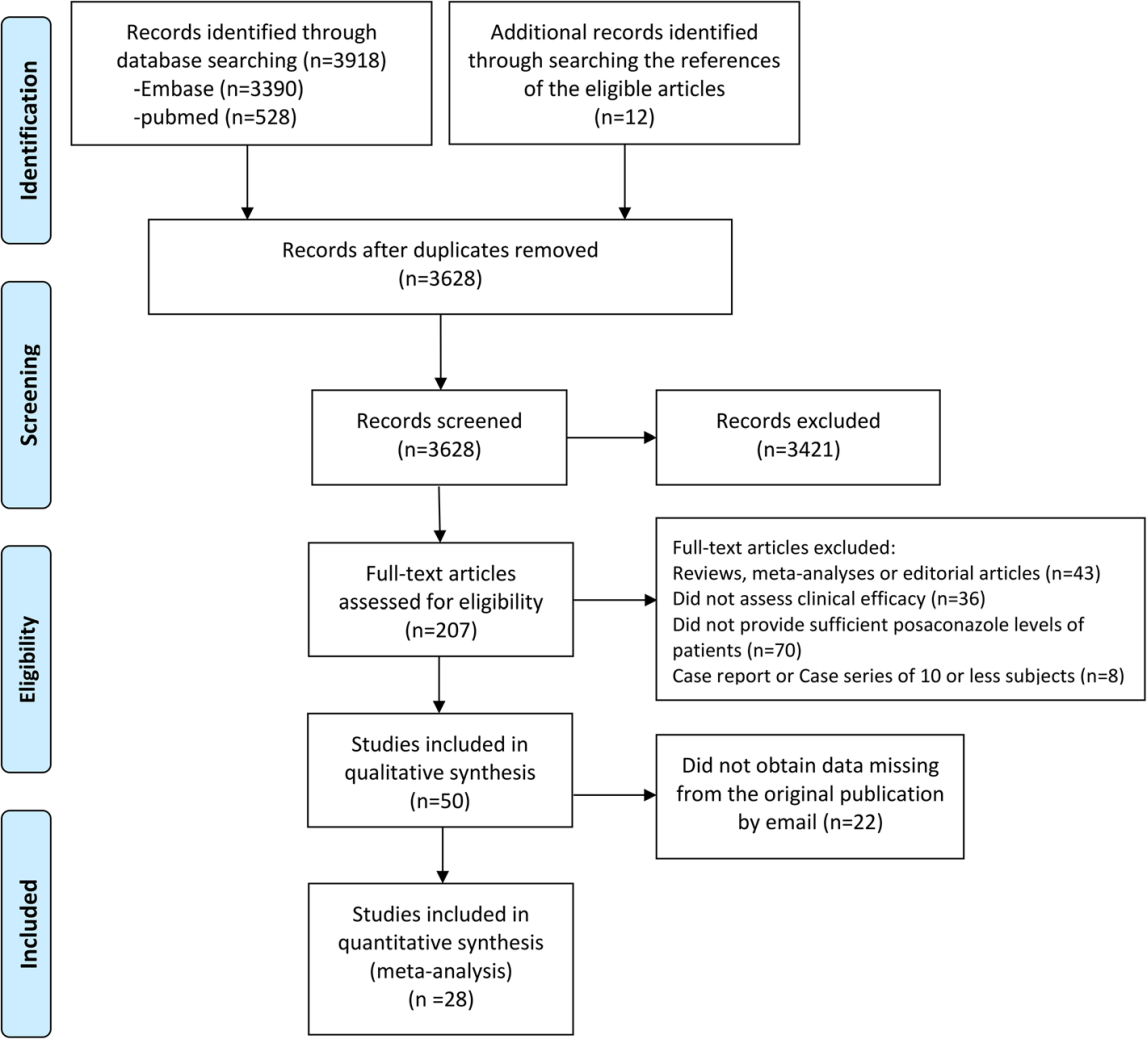
Flow diagram of study selection

Two reviewers (LC, YW) independently evaluated each study and identified whether they met the predefined inclusion criteria. The methods in Systematic Reviews and Meta-Analyses (PRISMA) criteria were used for the search and flow of studies (Fig. 1). Inclusion criteria for eligible study: (i) concerned patients who received posaconazole for prophylaxis of IFIs and reported posaconazole concentrations at steady state (≥7 days) [21]; (ii) evaluated clinical efficacy or toxicity; (iii) provided the incidence of successful response at a given cut-off value; (iv) was an original article (not meta-analysis, review or editorial article); (v) was not case reports or case series of sample size < 10 patients. (vi) used data derived from real patients rather than simulation results by models.

### Data extraction and quality assessment

A broad range of data was extracted from each study into a spreadsheet by two investigators (LC, YW) independently, including year of publication, author, country, study design, sample size, baseline characteristics, and cut-off value, outcomes of interest and safety events. Disagreement on the specific data between two reviewers was resolved by discussion, with planned involvement of a third investigator (TZ) if consensus was not achieved. We contacted the authors to obtain data missing from the original publication by email when required.

Cochrane Collaboration’s tool was applied to access the presence of sources of bias in randomized trials and the Newcastle–Ottawa Scale (NOS) was used for observational cohort and case–control studies [22, 23]. The NOS score ranges from 0 to 9, with higher score associated with better quality and low risk of bias. We reported the risk of bias summary for each item for studies included.

### Statistical analysis

We evaluated the exposure–response relationship between posaconazole plasma concentration and clinical efficacy for IFIs prevention. When the plasmas were measured at different sampling time, we chose the results measured near day 7 (≥7 days). The cut-off values that defined the therapeutic and subtherapeutic levels were extracted and depended on each individual study. For each cut-off value, we compared the successful rates of IFIs prevention among patients with subtherapeutic posaconazole levels and those with therapeutic levels. Failure of prevention was defined as the incidence of breakthrough IFIs including possible, probable or proven IFIs according to the European Organization for Research and Treatment of Cancer/Invasive Fungal Infections Cooperative Group and the National Institute of Allergy and Infectious Diseases Mycoses Study Group (EORTC/MSG) criteria [24]. The relationship between posaconazole concentration and clinical efficacy was assessed by OR values and confidence intervals. The Cochran Q χ^2^ test and I^2^ value were used to assess statistical heterogeneity, with a *P* > 0.1 an I^2^ value of less than 50% indicates a low level of heterogeneity.

We performed subgroup analysis to determine if the threshold results were influenced by some factors including population type (children and adults), underlying disease (cardiothoracic transplantation and hematological malignancy). The cut-off values used in subgroup analysis depended on the original thresholds provided in each individual study. Odds ratios (ORs) and 95% confidence intervals (CIs) were not be able to report if the number of studies in each subgroup less than 2 or when the outcomes of interest were not evaluated (both successful rates were 100% or 0% in group with subtherapeutic levels or therapeutic posaconazole levels). We also performed a sensitivity analysis to examine whether the main results were impacted by excluding a single study or by excluding several studies which was examined with a specific standard, such as NOS score ≤ 6, intervention following TDM results (intervention excluded), small sample size (*n* < 30 excluded), IFIs diagnosed following EORTC/MSG criteria (studies without specific criteria excluded).

Trial sequential analysis (TSA) was conducted to evaluate the reliability of the result using TSA software (version 0.9.5.10 Beta, Copenhagen Trial Unit) [25]. TSA performs a cumulative meta-analysis, which creates a Z curve of the summarized observed effect (the cumulative number of included patients and events) and the monitoring boundaries for benefit, harm, and futility, and the required information size (RIS, the sample size needed in a meta-analysis to detect or reject a certain intervention effect) [26–29]. TSA boundaries were constructed to assess the risk of random error when the number of available participants is less than the RIS and the potential necessity for repeated updates [30]. If the Z curve of the cumulative meta-analysis crosses any of the boundary (including the TSA, futility or RIS curve), no further studies are required, and there is sufficient information to support the conclusions. We assumed a type I error of 5% (two sides) and the power at 80% [27, 28].

A *P* value of < 0.05 (two-sided) was considered statistically significant. Statistical analyses were performed using RevMan version 5.3 and Stata version 12.0 (Statacorp LP, College Station, TX).

## Results

### Study selection and characteristics of included studies

Of the 3628 studies identified by the electronic and manual search, twenty-eight [9, 11, 12, 14, 15, 20, 21, 31–52] literatures were selected on the basis of inclusion criteria. The process of study selection is shown in Fig. 1. Table 1 summarized the characteristics of the final 28 studies included in assessing the exposure-response relationships between posaconazole levels and efficacy of IFIs prevention. Patients in most of studies are adults, with only 3 studies included exclusively pediatric patients [31, 48, 50]. Two new posaconazole formulations, tablet and injection, were reported in 5 and 2 studies [34, 36, 37, 50–52], respectively. Four studies received posaconazole both for prophylaxis and treatment while the remaining studies merely used posaconazole for prevention [14, 15, 40, 45]. The majority of studies included patients with hematological malignancy, followed by cardiothoracic transplantation and other underlying disease with a high risk of fungal infection.

**Table 1:** Characteristics of included studies

Study	Year	Country	Study design	Patients	Population	Main underlying disease (%)	POS form	Indication of therapy	Assay method	Intervention following TDM result	Prophylaxis duration: (days)	Definition of successful outcome	Cut-off value of prophylaxis	AE incidence
Sengar	2016	India	SCP	45	A	AML	sus	P	HPLC	No	NA	EORTC/MSG	Css ≥ 0.7	NA
Döring	2017	Germany	SCR	63	C	HSCT	sus & tab	P	HPLC	No	median 106	EORTC/MSG	Css ≥ 0.5	hepatotoxicity^d^
Liebenstein	2017	USA	SCR	74	A	AML	sus & tab	P	NA	Yes	31 & 34	EORTC/MSG	Css ≥ 0.7	hepatotoxicity (4.1%)
Tverdek	2017	USA	SCR	76	A	AML (55%)	tab & iv	P	NA	Yes	median 8	EORTC/MSG	Css ≥ 0.7	NA
Thakuria	2016	UK	SCP	26	A	lung transplant	sus	P	LC-MS/MS	Yes	36.1	EORTC/MSG	Css ≥ 0.5	8/27 (29.6%)
Vanstraelen	2016	Belgium	SCP	14	C	AML (50), ALL (36)	sus	P	HPLC	No	21 (17–60)	NA	Css ≥ 0.7	0
Park	2016	Korea	MCP	114	A	AML (91), MDS (9)	sus	P	LC-MS/MS	Yes	≥ 8	EORTC/MSG	Css ≥ 0.5	NA
Cornely	2016	Global(15 countries)	MCP, phase III	210 (Css is, available in 186)	NA	AML (54), MDS (3), HSCT (43)	tab	P	LC-MS/MS	No	≥ 28	EORTC/MSG	Css ≥ 0.5^a,^ Css ≥ 0.7^a^	84/210 (40%)
Hummert	2015	USA	SCR	29	A	AML	sus	P	NA	Yes	100	NA	Css ≥ 0.5, Css ≥ 0.7	0
Chae	2015	Korea	SCP	122	A	AML (94) + MDS (6)	sus	P	LC-MS/MS	No	25 (7–45)	EORTC/MSG	Css ≥ 0.5	NA
Maertens	2014	3 European countries	MCP, phase III	66	A	AML (94) + MDS (6)	inj	P	LC-MS/MS	No	1–14	EORTC/MSG	Css ≥ 0.5	200 mg/d, 44%, 300 mg/d, 33%
Duarte	2014	Western country	MCP, phase III	54	A	AML (91) + MDS (9)	tab	P	LC-MS/MS	No	NA, ≤ 28	EORTC/MSG	Css > 0.5	24/54 (44.4%)
Desplanques	2014	France	SCR	43	A	AML	sus	P	LC-MS/MS	No	NA	EORTC/MSG	Css > 0.5	NA
Bourdelin	2014	France	SCP	35	A + P	HM (AML 49%)	sus	P	HPLC	No	5–42	EORTC/MSG	Css ≥ 0.5	NA
Gross	2013	Germany	SCP	31 (27 P + 4 T)	A	AML (74), MDS (6)	sus	P + T	HPLC	No	NA	EORTC/MSG	Css ≥ 0.7	NA
Cattaneo	2013	Italy	SCP	50	NA	AML	sus	P	NA	No	NA	EORTC/MSG	Css ≥ 0.5	NA
Tonini	2013	France	SCR	29	A	HSCT	sus	P	LC-MS/MS	No	NA, ≥ 7	EORTC/MSG	Css ≥ 0.7	NA
Ross	2012	USA	SCP	54	A	AML (95), MDS (5)	sus	P	HPLC	No	NA, ≥ 7	EORTC/MSG	Css ≥ 0.5, Css ≥ 0.7	NA
Pavan	2012	Italy	SCR	50	NA	AML	sus	P	HPLC	Yes	NA	EORTC/MSG	Css ≥ 0.5	NA
Hoenigl	2012	Austria	SCP	34 (31 P + 3 T)	NA	AML/MDS (52), HSCT (48) for prophylaxis	sus	P + T	HPLC	No	NA	EORTC/MSG	Css ≥ 0.5	Hepatotoxicity (8.8%)
Eiden	2012	France	SCP	63 (50 samples on d7)	A	leukemia (48%), multiple myeloma (22)	sus	P	HPLC	No	median 14 (3–307)	EORTC/MSG	Css ≥ 0.5 ^b,^ Css ≥ 0.7 ^b^	Hepatotoxicity (6.3%)
Shields	2011	USA	SCR	17 (11 P + 6 T)	A	16 lung transplant	sus	P + T	HPLC	Yes	NA	EORTC/MSG	Css ≥ 0.5, Css ≥ 0.7	NA
Fanci	2011	NA	SCP	13	NA	AML	sus	P	HPLC	No	mean 23	NA	Css ≥ 0.5	NA
Bryant	2011	USA	SCR	21	A	AML (95) + MDS (5)	sus	P	HPLC	Yes	≥ 7	EORTC/MSG	Css ≥ 0.5, Css ≥ 0.7	NA
Welzen	2011	Netherlands	MCP, phase II	12	C	CGD	sus	P	HPLC	Yes	≥ 30	NA	Css ≥ 0.5^c,^ Css ≥ 0.7^c^	4/12 (33.3%)
Lebeaux	2009	France	SCR	54 (36 P + 18 T)	A	HM (69)	sus	P + T	HPLC	No	≥ 5	EORTC/MSG	Css ≥ 0.5	Hepatotoxicity (7.4%)
Ullmann & Jang	2007 & 2010	Global	RCT	291 P (Css is available in 252)	NA(> 95% adult)	HSCT	sus	P	LC-MS/MS	No	mean 80	EORTC/MSG	Css ≥ 0.7	107/301 (36%)
Cornely & Jang	2007 & 2010	Global	RCT	304 P (Css is available in 215)	NA	AML (84), MDS (16)	sus	P	LC-MS/MS	No	mean 29	EORTC/MSG	Css ≥ 0.7	19/304 SAE (6.2%)

*P* prophylaxis, *T* therapeutic, *Css* steady-state concentrations, *NA* not available, *SCR* single-center retrospective, *SCP* single-center prospective, *MCR* multicenter retrospective, *RCT* randomized controlled trial, *A* adult, *C* children, *HM* hematological malignancy, *AML* acute myeloid leukemia, *MDS* myelodysplastic syndrome, *HSCT* hematopoietic stem cell transplantation, *GVHD* graft versus host disease, *CGD* chronic granulomatous disease, *sus* suspension, *tab* delayed-release tablet, *inj* injection

^a^: concentration data gained on day 8

^b^: data selected from the day 7 based on 50 samples

^c^: data chosen from day 10

^d^: the rate of the hepatotoxicity differs from different standards

### Risk of bias

The study quality was evaluated independently by two investigators (LC and WY). Observational studies [9, 11, 14, 15, 31–52] were assessed for risk of bias using the NOS and were of moderate to high quality (Table 2). The Cochrane Collaboration’s tool was used to assess risk of bias of two randomized controlled trial studies [20, 21] (Additional file 1: Figure S1).

**Table 2:** Newcastle-Ottawa scoring of studies assessing efficacy

Study	Representativeness of the exposed cohort	Selection of the non-exposed cohort	Ascertainment of exposure	Outcome of intferest was not present at start	Comparability (score 0,1 or 2)	Assessment of outcome	Sufficient follow-up of outcome	Adequacy of follow up of cohorts	Score
Thakuria, L., et al.	NA	1	1	1	1	1	1	1	7/9
Vanstraelen, K., et al.	**1**	1	1	1	1	0	1	1	7/9
Park, W. B., et al.	1	1	1	1	1	1	NA	0	6/9
Cornely, O. A., et al.	1	1	1	1	2	1	1	1	9/9
Hummert, S. E., et al.	NA	1	0	1	1	0	1	1	5/9
Chae, H., et al.	1	1	1	1	1	1	1	1	8/9
Maertens, J., et al.	1	1	1	1	1	1	1	1	8/9
Duarte, R. F., et al.	1	1	1	1	1	NA	1	1	7/9
Desplanques, P. Y., et al.	NA	1	1	1	1	1	1	1	7/9
Bourdelin, M., et al.	1	1	1	1	1	1	1	1	8/9
Gross, B. N., et al.	1	1	1	1	1	1	1	1	8/9
Cattaneo, C., et al.	NA	1	0	1	1	NA	1	1	5/9
Tonini, J., et al.	1	1	1	1	1	1	1	1	8/9
Ross, A. L., et al.	NA	1	1	1	1	1	1	1	7/9
Pavan, L., et al.	NA	1	1	1	1	NA	1	1	6/9
Hoenigl, M., et al.	1	1	1	1	1	1	1	1	8/9
Eiden, C., et al.	1	1	1	1	0	1	1	1	7/9
Shields, R. K., et al.	1	1	1	1	1	1	1	1	8/9
Fanci, R., et al.	1	1	1	1	1	0	1	1	7/9
Bryant, A. M., et al.	NA	1	1	1	1	1	1	1	7/9
Welzen, M. E., et al.	1	1	1	1	1	0	1	1	7/9
Lebeaux, D., et al.	NA	1	1	1	1	1	1	1	7/9
Sengar	1	1	1	1	0	1	1	1	7/9
Döring	1	1	1	1	1	1	1	1	8/9
Liebenstein	1	1	NA	1	2	1	NA	1	7/9
Tverdek	1	1	NA	1	1	1	NA	1	6/9

*NA* not available

### Evaluation of prophylactic efficacy

All 28 [9, 11, 12, 14, 15, 20, 21, 31–52] studies, with 1930 enrolled patients, contributed to our systematic analysis of the relationship between posaconazole plasma concentration and rate of clinical prophylaxis success. Twenty participating studies [9, 11, 14, 15, 32–39, 41, 43–48, 50], with 1043 patients, provided outcomes of interest at a cut-off value of 0.5 mg/L; and 15 studies [9, 12, 20, 21, 31, 34, 35, 40, 42, 43, 45, 47–49, 51, 52], with 1098 patients, provided data at a cut-off value of 0.7 mg/L.

**Fig. 2:**
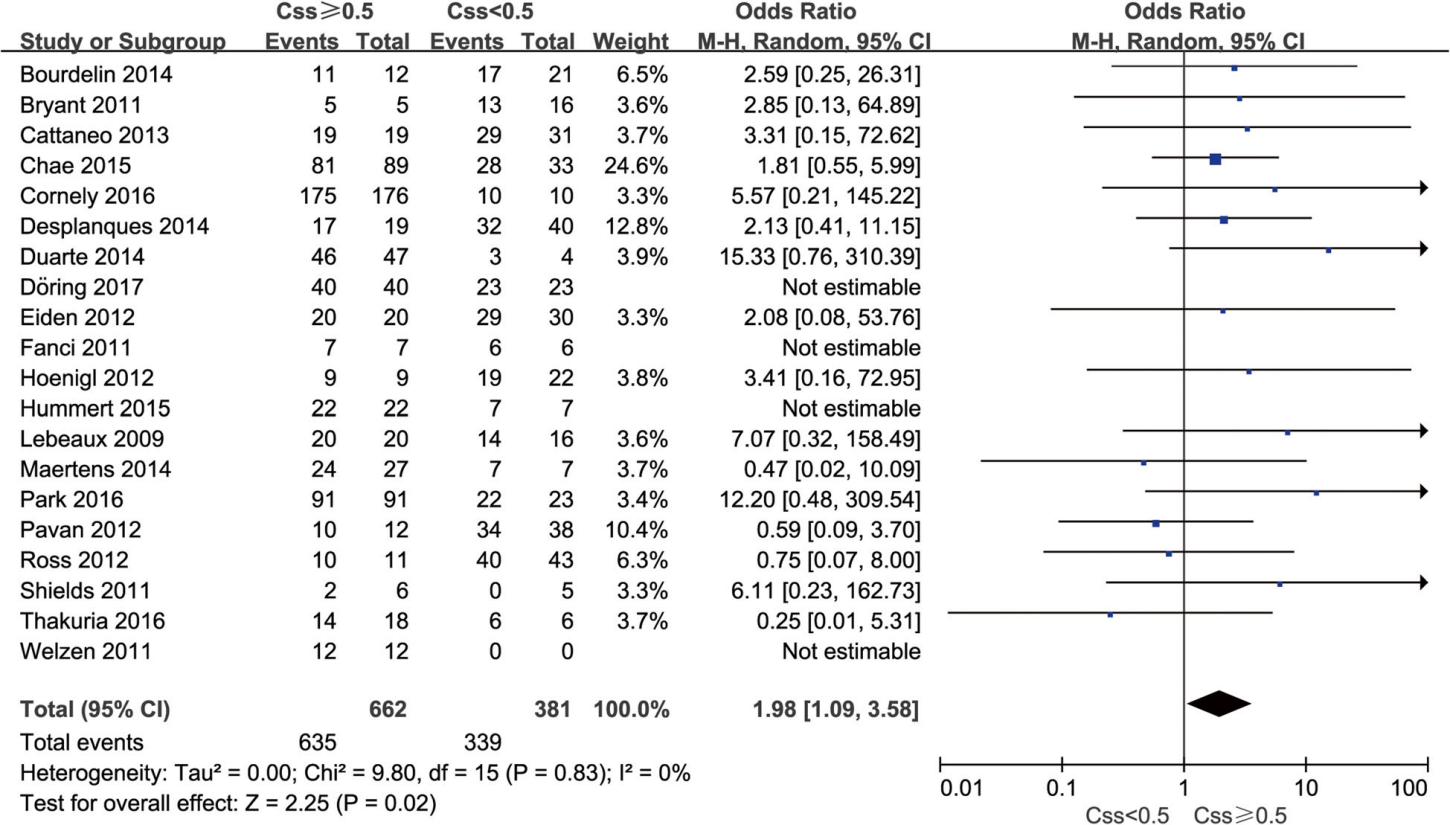
Meta-analysis for successful outcome among patients with steady-state posaconazole plasma concentration ≥ 0.5 mg/L compared with patients with < 0.5 mg/L

The overall pooled rate of successful prevention was 88.2% among 28 enrolled studies. The prophylactic threshold value was defined by each individual study at 0.5 and 0.7 mg/L. As shown in Fig. 2, there was a significant difference at the cut-off value of 0.5 mg/L at which successful prophylactic outcome was achieved among 95.9% of patients with posaconazole concentrations ≥0.5 mg/L compared with 89.0% of those with concentrations < 0.5 mg/L (*P* = 0.02). Patients with posaconazole plasma concentrations ≥0.5 mg/L had a significant chance of successful prophylaxis against IFIs about 2-fold that of patients with concentrations < 0.5 mg/L (OR = 1.98, 95% CI 1.09–3.58, I^2^ = 0%).

**Fig. 3:**
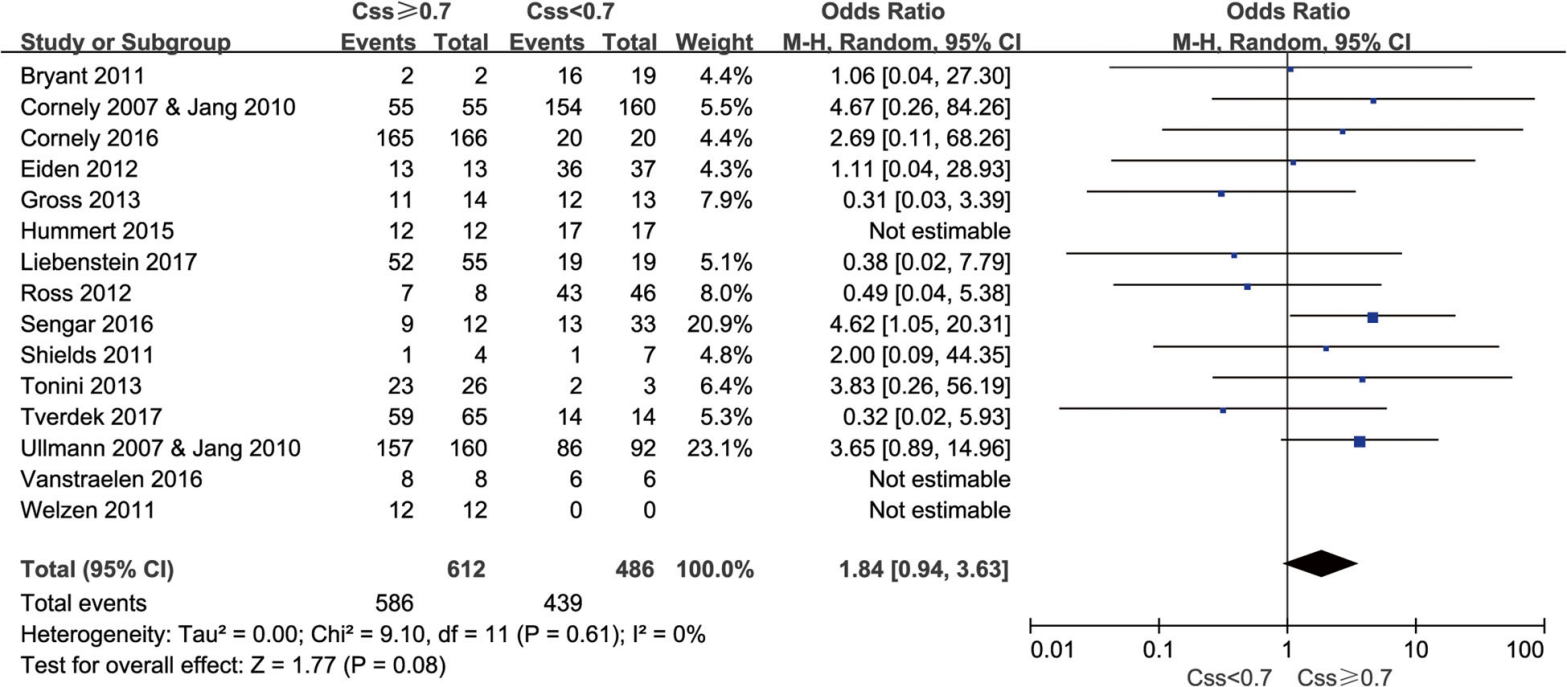
Meta-analysis for successful outcome among patients with steady-state posaconazole plasma concentration ≥ 0.7 mg/L compared with patients with < 0.7 mg/L

Among studies reported at the cut-off value of 0.7 mg/L, patients with posaconazole concentrations ≥0.7 mg/L had a rate of successful prophylactic outcome similar to that of those with concentrations < 0.7 mg/L (95.8% versus 90.3%) (*P* = 0.08) (Fig. 3).

No statistically significant difference was observed at this threshold although OR value was calculated as 1.84 (95% CI 0.94–3.63, I^2^ = 0%).

### Subgroup analysis

A summary of subgroup analysis for prophylactic efficacy was shown in Table 3. Three studies included pediatric patients but the successful rates appeared to be 100% or 0% which make the OR values not estimable between two research groups [31, 48, 50]. Thus, the predesigned subgroup of population type (children and adults) could not be analyzed due to limited relevant research.

**Table 3:** Summary of subgroup analysis for prophylaxis efficacy

Subgroup		Cut-off value(mg/L)	OR(95% CI)	No. of studies	No. of participants in experimental group	No. of participants in control group	I^2^%	P
Underlying disease	Cardiothoracic transplant	Css ≥ 0.5 vs. Css < 0.5	1.16 [0.05, 26.94]	2	16/24	6/11	49	0.92
	Hematological malignancy	Css ≥ 0.5 vs. Css < 0.5	2.06 [1.12, 3.82]	18	619/638	333/370	0	0.02
Underlying disease	Cardiothoracic transplant	Css ≥ 0.7 vs. Css < 0.7	2.00 [0.09, 44.35]	1	1/4	1/7	NA	0.66
	Hematological malignancy	Css ≥ 0.7 vs. Css < 0.7	1.84 [0.92, 3.68]	14	585/608	438/479	0	0.09

*NA* not available

For the cut-off value of 0.5 mg/L, successful outcomes of patients with hematological malignancy presented significant difference among those with therapeutic and subtherapeutic levels while there was no statistical significance for cardiothoracic transplant recipients (34 lung transplantations and 1 heart transplantations) (*P* = 0.02 and *P* = 0.97, respectively). For cut-off value of 0.7 mg/L, neither group showed a significant difference (*P* = 0.09 and *P* = 0.66, respectively).

### Publication bias and sensitivity analysis

**Fig. 4:**
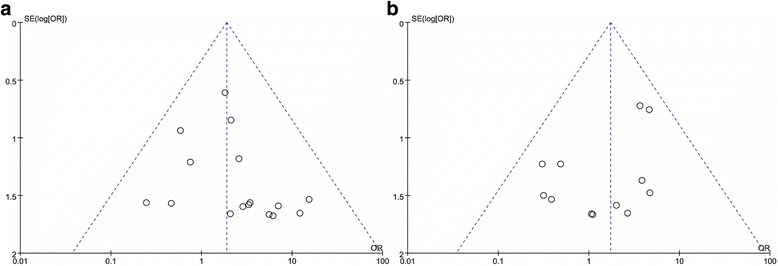
Funnel plots for the cut-off value of 0.5 mg/L (**a**) and 0.7 mg/L (**b**)

We evaluated publication bias at the steady-state concentration cut-off value of 0.5 mg/L (20 studies) and 0.7 mg/L 15 studies) prophylaxis. The results of funnel plots seemed asymmetric under cut-off value of 0.5 mg/L. (Fig. 4). However the Harbord’s test (*P* = 0.16 and 0.26 under cut-off value of 0.5 and 0.7 mg/L) showed a low likelihood of publication bias.

Results of sensitivity analysis showed that the main results of meta-analysis were relatively stable after excluding each single study enrolled under both cut-off values (Additional file 1: Figure S2). Similarly, no change in effect was found when studies were analyzed by NOS score ≤ 6 [33, 35, 41, 44, 52], intervention following TDM results (intervention excluded) [32, 33, 35, 44, 45, 47, 48, 51, 52], small sample size (*n* < 30 excluded) [31, 32, 35, 42, 45–48], and IFIs diagnosed following EORCT/MSG criteria (studies without specific criteria excluded) [31, 35, 46, 48], except for a significant change after excluding studies from Liebenstein [51] and Tverdek [52] (Additional file 1: Table S1).

### Trial sequential analysis

**Fig. 5:**
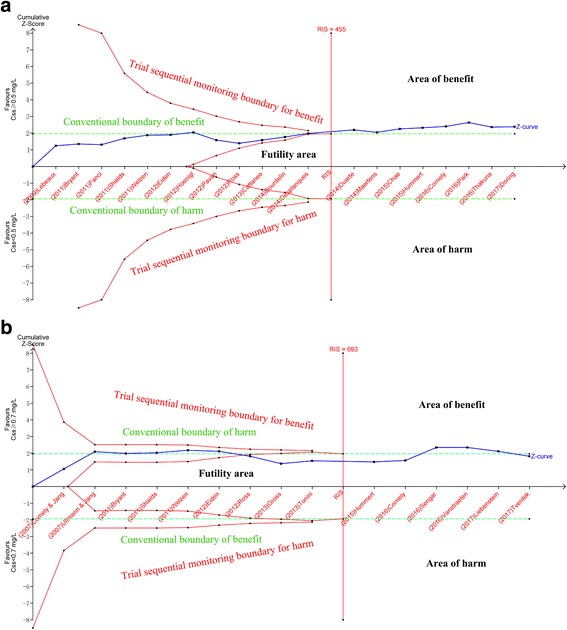
The results of trial sequential analysis under two posaconazole TDM targets. **a** Trial sequential analysis in 20 trials for posaconazole concentration target of 0.5 mg/L. The required information size (RIS, i.e., number of participates) was calculated as 455. The Z curve crossed the conventional boundary of benefit and the vertical line of RIS. **b** Trial sequential analysis in 15 trials for posaconazole concentration target of 0.7 mg/L. The RIS was calculated as 693. The Z curve crossed the futility boundary and the vertical line of RIS

TSA was performed in our study to analyze whether the available data were powered enough to reach firm conclusions in the present study. For the posaconazole concentration target of 0.5 mg/L, TSA showed that it could benefit more on the prevention successful rate when a posaconazole level is more than 0.5 mg/L, as the number of patients evaluated for this breakpoint (*n* = 1043) surpassed the optimal sample sizes (*n* = 455) (Fig. 5a). Although there was some fluctuation before the Z curve reaching the RIS, the Z curve remained outside of the conventional benefit boundary after crossing the RIS. For the posaconazole target of 0.7 mg/L, TSA showed that patients with posaconazole level over 0.7 mg/L did not show significant priority on the successful prevention rate comparing with those with lower than 0.7 mg/L. With the all the available data included, the number of patients evaluated for this breakpoint (*n* = 1098) also surpassed the optimal sample sizes (*n* = 693) for the same outcome (Fig. 5b). However, it should be noted that the Z curve turned to be out of the conventional boundary after adding three recent studies in our analysis [31, 49, 51].

## Discussion

This meta-analysis was designed to assess the exposure-response relationship between the reported posaconazole concentration and clinical prophylactic efficacy. Our pooled analysis demonstrated that a steady-state posaconazole target of ≥0.5 mg/L is more predictive for successful prophylaxis than the target of ≥0.7 mg/L. The results indicated that with posaconazole levels at 0.5 mg/L or higher, patients with hematological malignancies were twice more likely to achieve a successful prophylaxis.

We considered possible IFI as failed prevention because patients are usually enrolled in empirical treatment programs once they were diagnosed as possible IFIs. An ideal prophylaxis should prevent even a possible IFI result which could probably degenerate into probable or even proven and cause more medical costs and prolonged hospitalizations [5, 6]. Before our pooled analysis, there were two putative targets of steady state plasma, 0.5 mg/L and 0.7 mg/L, which were suggested as threshold levels for IFIs prophylaxis. The first target of 0.5 mg/L is based on the MIC_90_ for most *Aspergillus species* [23] and the second target of 0.7 mg/L, is considered the efficacy threshold by the FDA. [24] The evidence of the recommendation from FDA is based on a RCT trial in high risk patients with graft versus host disease (GVHD) after allogeneic hematopoietic stem cell transplantation (allo-HSCT) and the results showed that the successful prophylactic rate would be 98.1% when patients’ posaconazole average concentration reached over 0.7 mg/L, which is similar to our outcome (95.8%) [12, 21]. Ullmann et al. used the average posaconazole concentrations while most of our data were from trough concentrations which are more available in clinical practice [21]. As usual, the posaconazole average concentration is higher than the trough concentration although there is no significant fluctuation after steady state [19]; thus, it is reasonable that an average level of 0.7 mg/L could be considered as the threshold in Jang’s study and a lower trough level at 0.5 mg/L in our analysis [12].

It has been reported that IFIs are associated with high morbidity and mortality in patients with hematological malignancy [5, 6, 14]. Our study demonstrated that performing TDM could help posaconazole concentrations reach the threshold 0.5 mg/L and then improve the successful rate from 89.0% (95% CI 87.4%–90.6%) to 95.9% (95% CI 95.2%–96.7%) for prophylactic usage. The successful rate will also be improved from 90.3% (95% CI 89.0%–91.7%) to 95.8% (95% CI 94.9%–96.6%) if the posaconazole concentration reached at 0.7 mg/L or higher, yet there was no statistical significance. The possible explanation of this result is that patients with posaconazole stable concentration at the range of 0.5–0.7 mg/L were mostly identified as a successful prevention; thus, the target of 0.7 mg/L was evaluated with no significance. Additionally, it has been proved that posaconazole concentrations in pulmonary alveolar cells are over 40-fold higher than those in plasma [53]. So the high posaconazole aggregation pulmonary could be explicable for achieving satisfactory effects under low posaconazole plasma levels.

For the result of subgroup analysis based on the underlying disease, a target of 0.5 mg/L on the steady state is recommended in patients with hematological malignancies but not for cardiothoracic transplant recipients. There was only one study involved in the solid-organ transplant group at the target of 0.7 mg/L, so whether 0.7 mg/L could be a target of this cardiothoracic transplant population still need further studies to confirm. Our results showed that the prophylactic efficacy have no significant difference when patients’ posaconazole levels were over or below 0.7 mg/L in cardiothoracic transplant recipients. The chief reasons are that posaconazole concentrations of this population showed great variability [32] and studies on this population are limited at present. Therefore, the utility of TDM in cardiothoracic transplant recipients and the specific posaconazole target warrant further investigation.

Both heterogeneity and the publication bias in our pooled analysis are low. Sensitivity analysis was done by exclusion of studies with NOS ≤ 6, intervention following TDM results and small sample size with *n* < 30. It shows that the significance of main outcome at 0.5 mg/L remained stable after excluding studies mentioned above or removing each individual study, which confirmed the high reliability and stability of our meta-analysis. However, after excluding 6 studies following TDM intervention [35, 45, 47, 48, 51, 52], the insignificant outcome (*P* = 0.08) of the target at 0.7 mg/L turned into significant (*P* = 0.03). This change might attribute to the influence of dosing adjustment, which could interfere the standard-compliant samples or the prevention outcome.

A particular section of our meta-analysis was the result verification using of TSA. According to the TSA results, it is noticeable that the Z curves were not stable with the growing data. For the TSA result of 0.7 mg/L, the Z curve escaped out of the conventional boundary even after reaching the optimal sample size. Study from Sengar was supposed to be the main reason contributing to this reverse change because the Z curve turned back to the conventional boundary after adding the other two studies (Vanstraelen and Liebenstein) into the analysis. However, only the abstract is available in Sengar’s study and maybe the race of the participants (Asian) could be a possible reason to explain this result. In brief, the TSA results under two posaconazole concentration targets indicated that there are sufficient information to support our conclusion. However, only two RCTs are available in our study, a well-designed prospective trial is needed to verify our results.

According to the involved studies, we found that posaconazole concentration is higher when patients are administered with those two new formulations, delayed-release tablet and injection formulation due to the stable bioavailability [5]. Thus, the number of patients with subtherapeutic levels is less and there were five and two studies involved in groups of tablet and injection in our analysis, respectively. Since posaconazole delayed-release tablet and the injection forms could increase the possibility of achieving the target, whether TDM is useful in this case still needs future investigation with large sample size. To date, posaconazole oral suspension formulation is still widely used and remains available worldwide, and this form is still an important option for patients with nasogastric tubes or those unable to take tablets [11]. Therefore, the target of 0.5 mg/L is required and should be recommended during the TDM process for patients administered with posaconazole oral suspension.

It was reported that posaconazole showed a good safety profile during a standard long-term administration. The most common adverse event which related to the treatment is gastrointestinal tract disturbances [20, 54, 55] and the incidence of serious adverse events is 6% - 13%, including the hepatotoxicity, QTc prolongation, etc. [20, 21]. Thus, studies about the relationship between posaconazole exposure and adverse events are still limited, which make it not viable to explore the threshold for safety concentration like voriconazole [56, 57]. To date, posaconazole appears to have a more favorable safety and tolerability profile than voriconazole [58, 59]. Only a few studies accessed the relationship between posaconazole exposure and treatment efficacy, which make it infeasible to define a posaconazole concentration target by meta-analysis. Walsh et al. conducted a study in which a cohort of 67 patients who received posaconazole for salvage treatment of invasive *Aspergillosis*, the results demonstrated that the cure rates increased with growing posaconazole average concentration quartiles. The cure rates could achieve 75% when posaconazole average concentration reached at 1.25 mg/L; thereby this quartile value was subsequently accepted as a threshold for IFIs treatment. Further research like a prospective and well-powered study is required to investigate the optimum posaconazole concentration for ensuring safety of posaconazole and efficacy of salvage therapy.

### Strengths and weaknesses

Our study has several strengths. This is the first pooled analysis comparing two commonly used but disputed cut-off values for prophylactic efficacy of posaconazole and the results recommended an optimal target for posaconazole usage in IFIs prevention. Besides, we implemented subgroup analysis to seek the suitable targets for patients in different underlying diseases. Our results recommended 0.5 mg/L as a target concentration for IFIs prophylaxis in patients with hematological malignancy, which is more likely to achieve than 0.7 mg/L; hence, it may help to reduce the posaconazole dosage and financial burden for patients and simultaneously ensure the prophylactic efficacy in the long-term usage of posaconazole.

Potential limitations of our study merit discussion. First, we did not investigate the relationship between treatment efficacy, safety and posaconazole exposure owing to the limited number of published studies. Second, survival benefit on each cut-off values have not been explored due to the low mortality and short follow-up time of studies involved. Third, studies concerning the direct comparison of the clinical outcomes of patients taking posaconazole for prophylaxis of IFI with and without TDM are limited, so we are not able to validate the practical benefit of posaconazole TDM in clinical up to date. Further studies are needed in this respect. Finally, the inevitable limitation of all meta-analysis is that the quality of the results are directly related to the quality of individual studies included in the analysis. Except for two randomized controlled trials, all were cohort studies, many of which used a small sample size and focused on a single center. However, we provide the largest pooled analysis of the relationship between posaconazole TDM and clinical efficacy of IFIs prevention. The present study highlights high quality studies in this area is poor and emphasizes the remaining controversy regarding the relationship between posaconazole TDM and treatment efficacy and safety. A well-designed prospective trial to assess the utility of posaconazole TDM, especially in reference to survival, successful response and toxicity, is warranted.

## Conclusion

TDM of posaconazole prophylaxis with the oral suspension has been increasingly used and therefore recommendations regarding target plasma are urgent needed. Based on the results from our meta-analysis, we conclude that patients with posaconazole plasma concentrations ≥0.5 mg/L are associated with an increased probability of successful IFIs prevention exclusively for those with hematological malignancies. Our study highlights the lack of the studies regarding the relationship between TDM and clinical outcome in the newly released formulations: tablet and injection of posaconazole.

## Additional file

Additional file 1: **Table S1.** Summary of sensitive analysis for prophylaxis efficacy. **Figure S1.** Source of bias in two randomized trials following Cochrane Collaboration’s tool. **Figure S2.** Sensitive analysis of individual study involved at cut-off value of 0.5 mg/L (A) and 0.7 mg/L (B). Statement for detailed searching strategy. (PDF 301 kb)

## Abbreviations

A: Adult
allo-HSCT: Allogeneic hematopoietic stem cell transplantation
AML: Acute myeloid leukemia
C: Children
CGD: Chronic granulomatous disease
CI: Confidence interval
Css: steady-State concentrations
ECIL: European Conference on Infections in Leukaemia
EORTC/MSG: European Organization for Research and Treatment of Caner/Invasive Fungal Infections Cooperative Group and the National Institute of Allergy and Infectious Diseases Mycoses Study Group
ESCMID: European Congress of Clinical Microbiology and Infectious Diseases
FDA: Food and Drug Administration
GVHD: Graft versus host disease
HM: Hematological malignancy
IDSA: Infectious Diseases Society of America
IFIs: Invasive fungal infections
inj: Injection
MCR: Multicenter retrospective
MDS: Myelodysplastic syndrome
NA: Not available
NOS: Newcastle–Ottawa Scale
OR: Odds ratio
P: Prophylaxis
RCT: Randomized controlled trial
RIS: Required information size
SCP: Single-center prospective
SCR: Single-center retrospective
sus: Suspension
T: Therapeutic
tab: Delayed-release tablet
TDM: Therapeutic drug monitoring
TSA: Trial sequential analysis
