# Supplementary material for: Utility of posaconazole therapeutic drug monitoring and assessment of plasma concentration threshold for effective prophylaxis of invasive fungal infections: a meta-analysis with trial sequential analysis
Source: BMC Infect Dis. 2018 Apr 2;18:155. doi: 10.1186/s12879-018-3055-3 (PMC5879730; doi:10.1186/s12879-018-3055-3)
Supplement: Supplementary file 1 — Table S1. Summary of sensitive analysis for prophylaxis efficacy. Figure S1. Source of bias in two randomized trials following Cochrane Collaboration’s tool. Figure S2. Sensitive analysis of individual study involved at cut-off value of 0.5 mg/L (A) and 0.7 mg/L (B). Statement for detailed searching strategy. (PDF 301 kb) [file 12879_2018_3055_MOESM1_ESM.pdf]

Table SI. Summary of sensitive analysis for prophylaxis efficacy

Term excluded	Studies excluded	Cut-off value(mg/L)	OR(95% CI)-primary random effect model	OR(95% CI)-after removing random effect model	P-primary	P- after removing
NOS ≤ 6	Park 2016	Css≥0.5	1.98 [1.09, 3.58]	2.09 [1.09, 4.01]	0.02	0.03
	Hummert 2015					
	Cattaneo 2013					
	Pavan 2012					
	Tverdek 2017	Css≥0.7	1.84 [0.94, 3.63]	2.04 [1.01, 4.09]	0.08	0.05
	Hummert 2015					
Intervention following TDM result (intervention excluded)	Thakuria 2016	Css≥0.5	1.98 [1.09, 3.58]	2.23 [1.13, 4.42]	0.02	0.02
	Park 2016					
	Hummert 2015					
	Pavan 2012					
	Shields 2011					
	Bryant 2011					
	Welzen 2011					
	Liebenstein 2017	Css≥0.7	1.84 [0.94, 3.63]	2.35 [1.10, 4.99]	0.08	0.03
	Tverdek 2017					
	Hummert 2015					
	Shields 2011					
	Bryant 2011					
	Welzen 2011					
small sample size (n<30 excluded)	Thakuria 2016	Css≥0.5	1.98 [1.09, 3.58]	2.04 [1.09, 3.82]	0.02	0.03
	Hummert 2015					
	Bryant 2011					
	Fanci 2011					
	Welzen 2011					
	Shields 2011					
	Vanstraelen 2016	Css≥0.7	1.84 [0.94, 3.63]	1.69 [0.77, 3.71]	0.08	0.19
	Hummert 2015					
	Tonini 2013					
	Bryant 2011					
	Welzen 2011					
	Shields 2011					
IFI diagnosed following EORCT/MSG criteria (studies without specific criteria excluded)	Hummert 2015	Css≥0.5	1.98 [1.09, 3.58]	1.98 [1.09, 3.58]	0.02	0.02
	Fanci 2015					
	Welzen 2011					
	Vanstraelen 2016	Css≥0.7	1.84 [0.94, 3.63]	1.84 [0.94, 3.63]	0.08	0.08
	Hummert 2015					
	Welzen 2011					

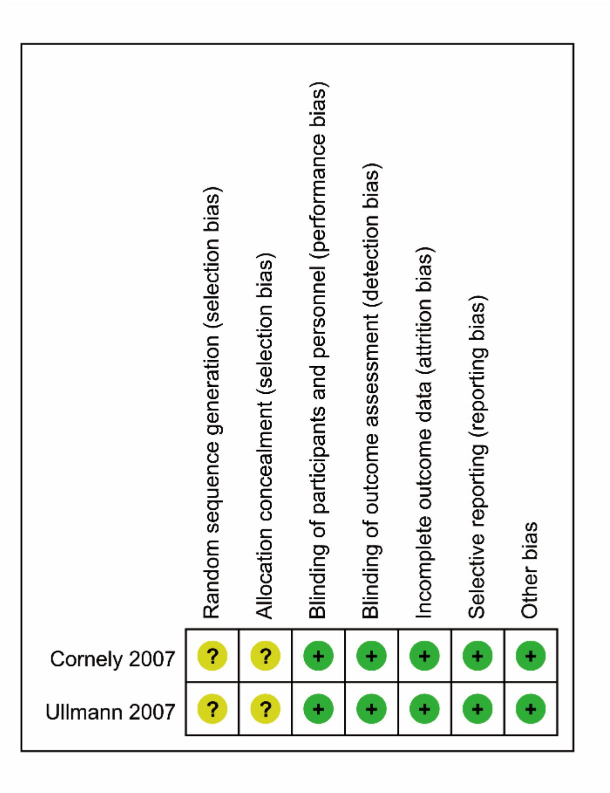

Figure S1. Source of bias in two randomized trials following Cochrane Collaboration's tool.

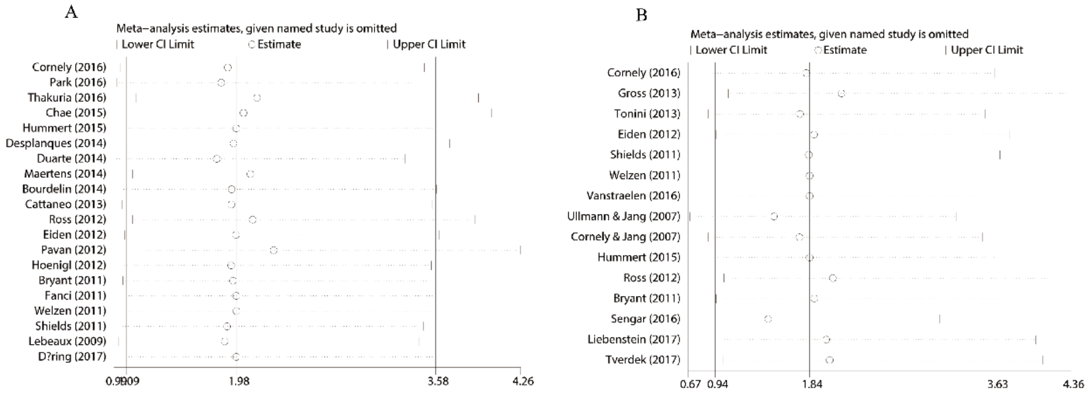

Figure S2. Sensitive analysis of individual study involved at cut-off value of 0.5 mg/L (A) and 0.7 mg/L (B).

Statement for detailed searching strategy

Two database, PubMed and EMBASE were searched before September 1, 2017. The search from PubMed and EMBASE were all limited to human studies.

PubMed (Search date: September 1, 2017)

((((("Drug Monitoring"[Mesh]) OR "Pharmacovigilance"[Mesh]) OR ("Drug-Related Side Effects and Adverse Reactions"[Mesh] OR "Adverse Drug Reaction Reporting Systems"[Mesh] OR "Drug Eruptions"[Mesh]))) OR ((((concentration) OR exposure) OR efficacy) OR response)) AND (((posaconazole) OR Noxafil) AND Humans[Mesh]))

Search result: 528

EMBASE (Search date: September 1, 2017)

#1 posaconazole OR noxafil AND [humans]/lim (5638)

#2 concentration OR exposure OR efficacy OR response (6,315,359)

#3 'drug monitoring'/exp OR 'drug surveillance program'/exp OR 'adverse drug reaction'/exp OR 'drug fatality'/exp (539,892)

#4 #2 OR #3 (6,612,498)

#5 #1 AND #4 (3390)

Search result: 3390
